# Bis(2,2′-bipyridine)(5-isothio­cyanato-1,10-phenanthroline)ruthenium(II) bis­(hexa­fluoridophosphate) acetonitrile solvate

**DOI:** 10.1107/S1600536809035302

**Published:** 2009-09-09

**Authors:** Samik Nag, Amlan K. Pal, Garry S. Hanan

**Affiliations:** a Département de Chimie, Université de Montréal, CP 6128, Succ. Centre-ville, Montréal, Québec, Canada H3C 3J7

## Abstract

The title compound, [Ru(C_10_H_8_N_2_)_2_(C_13_H_7_N_3_S)](PF_6_)_2_·CH_3_CN, was synthesized by the reaction of thio­phosgene and bis­(2,2′-bipyridine)(1,10-phenanthrolin-5-amine)ruthenium(II) bis­(hexa­fluoridophosphate). The Ru^II^ atom adopts a slightly distorted octa­hedral RuN_6_ coordinaton formed by four N atoms of two bipyridine ligands and by two N atoms of the 1,10-phenantroline ligand. The isothio­cyanate group is almost linear, with an N—C—S angle of 174.4 (6)°. Two of the three hexa­fluoridophosphate counter-anions are located on inversion centres.

## Related literature

The title compound was previously synthesized by two other groups (Ryan *et al.*, 1992 ▶; Khimich *et al.*, 2007 ▶). However, the crystal structure was not reported at that time. For the crystal structures of related compounds, see: Ye *et al.* (1999 ▶); Huang & Ogawa (2006 ▶); Batey *et al.* (2007 ▶). For the importance and applications of Ru^II^ complexes with bipyridine ligands, see: Bertini *et al.* (1994 ▶); Medlycott & Hanan (2005 ▶, 2006 ▶).
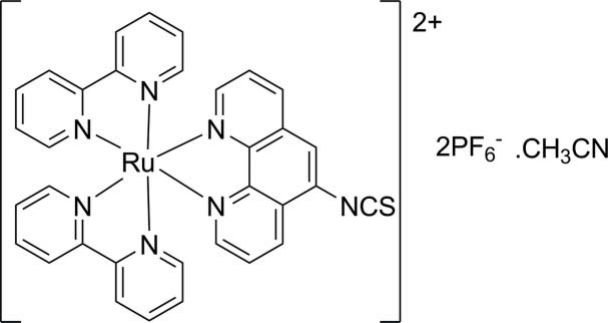


## Experimental

### 

#### Crystal data

[Ru(C_10_H_8_N_2_)_2_(C_13_H_7_N_3_S)](PF_6_)_2_·C_2_H_3_N
*M* *_r_* = 981.71
Triclinic, 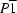

*a* = 9.129 (3) Å
*b* = 12.397 (4) Å
*c* = 17.084 (5) Å
α = 88.618 (4)°
β = 89.704 (5)°
γ = 72.870 (4)°
*V* = 1847.1 (10) Å^3^
*Z* = 2
Mo *K*α radiation
μ = 0.67 mm^−1^
*T* = 150 K
0.24 × 0.13 × 0.10 mm

#### Data collection

Bruker APEXII CCD diffractometer
Absorption correction: multi-scan (*SADABS*; Sheldrick, 1996 ▶) *T*_min_ = 0.83, *T*_max_ = 0.95
178384 measured reflections
6687 independent reflections
6155 reflections with *I* > 2σ(*I*)
*R*_int_ = 0.061

#### Refinement

*R*[*F* ^2^ > 2σ(*F* ^2^)] = 0.055
*wR*(*F* ^2^) = 0.152
*S* = 1.18
6687 reflections
536 parameters
H-atom parameters constrained
Δρ_max_ = 1.45 e Å^−3^
Δρ_min_ = −0.86 e Å^−3^

### 

Data collection: *APEX2* (Bruker, 2008 ▶); cell refinement: *SAINT* (Bruker, 2008 ▶); data reduction: *SAINT*; program(s) used to solve structure: *SHELXS97* (Sheldrick, 2008 ▶); program(s) used to refine structure: *SHELXL97* (Sheldrick, 2008 ▶); molecular graphics: *SHELXTL* (Sheldrick, 2008 ▶); software used to prepare material for publication: *UdMX* (Maris, 2004 ▶).

## Supplementary Material

Crystal structure: contains datablocks I, global. DOI: 10.1107/S1600536809035302/wm2252sup1.cif

Structure factors: contains datablocks I. DOI: 10.1107/S1600536809035302/wm2252Isup2.hkl

Additional supplementary materials:  crystallographic information; 3D view; checkCIF report

## Figures and Tables

**Table 1:** Selected bond lengths (Å)

Ru—N1	2.056 (4)
Ru—N3	2.059 (4)
Ru—N2	2.059 (4)
Ru—N4	2.064 (4)
Ru—N6	2.065 (4)
Ru—N5	2.072 (4)

**Fig. 1.**
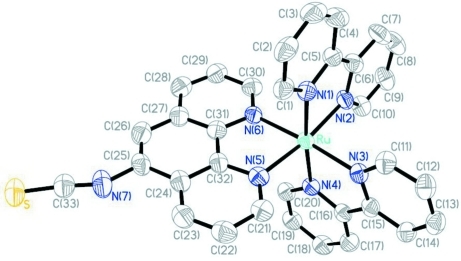
View of the cation in the title compound. Thermal ellipsoids are shown at the 50% probability level. H atoms, hexafluoridophosphate anions and the acetonitrile solvent molecule have been omitted for clarity.

## supplementary crystallographic information

### Comment

Ru^II^ polypyridyl complexes, mainly [Ru(bpy)_3_]^2+^ (bpy = 2,2'-bipyridine) and its derivatives have been studied extensively during the past three decades for their excellent photophysical properties characterized by long excited state lifetimes which makes them suitable choice for chromophores in light-harvesting devices (Medlycott & Hanan, 2005, 2006). Such good emissive properties of this class of compounds also make them useful in labelling biomolecules (Bertini *et al.*, 1994). The title compound can be used to label biomolecules through a thiourea linkage by reaction of the isothiocyanate group on it with an amino group of the protein (Ryan *et al.*, 1992; Khimich *et al.*, 2007).

The crystal structure of the title compound reveals that the ruthenium atom has a slightly distorted octahedral coordinaton sphere and the isothiocyanate group is almost linear with a N—C—S angle of 174.4 (6)°. The six Ru—N bond distances falls in a short range of 2.056 (4) to 2.072 (4) Å, with the two longer ones being that with the phenanthroline moiety. The bite angles for the 2,2'-bipyridine lingands are 79.03 (16)° and 78.91 (15)°, while that for the phenanthroline is 78.91 (15)°.

For crystal structures of related Ru^II^ complexes with bidentate polypyridyl ligands, see: Ye *et al.* (1999); Huang & Ogawa (2006); Batey *et al.* (2007).

### Experimental

Bis(2,2'-bipyridine)(1,10-phenanthroline-5-amine)ruthenium(II) bis(hexafluoridophosphate) (450 mg, 0.5 mmol) was dissolved in acetone (100 ml). To this dark red solution was added Na_2_CO_3_ (212 mg, 2.0 mmol). After addition of CSCl_2_ (0.5 ml) to the reaction mixture under N_2_ atmosphere, it was stirred for 7 h. Then the solvent was evaporated under reduced pressure and the residue dissolved in dichloromethane and filtered. The filtrate was dried over anhydrous Na_2_SO_4_. After removal of the solvent under reduced pressure, a red crystalline compound was obtained. Yield: 446 mg (95%). Red crystals suitable for X-ray crystallography were grown by slow diffusion of isopropyl ether into a dilute acetonitrile solution of the title compound.

### Refinement

The H atoms were positioned geometrically (aromatic C—H: 0.95 Å, methyl C—H: 0.98 Å) and were included in the riding model approximation; their temperature factors were set to 1.2 times those of the equivalent isotropic temperature factors of the parent site. The highest remaining electron density peaks are close to the Ru atom, with distances of 0.84 and 0.94 Å, respectively.

### Crystal data

**Table:**

[Ru(C_10_H_8_N_2_)_2_(C_13_H_7_N_3_S)](PF_6_)_2_·C_2_H_3_N	*Z* = 2
*M**_r_* = 981.71	*F*(000) = 980
Triclinic, *P*1	*D*_x_ = 1.765 Mg m^−^^3^
Hall symbol: -P 1	Mo *K*α radiation, λ = 0.71073 Å
*a* = 9.129 (3) Å	Cell parameters from 32297 reflections
*b* = 12.397 (4) Å	θ = 2.3–24.9°
*c* = 17.084 (5) Å	µ = 0.67 mm^−^^1^
α = 88.618 (4)°	*T* = 150 K
β = 89.704 (5)°	Block, red
γ = 72.870 (4)°	0.24 × 0.13 × 0.10 mm
*V* = 1847.1 (10) Å^3^	

### Data collection

**Table:**

Bruker APEXII CCD diffractometer	6687 independent reflections
Radiation source: X-ray sealed tube	6155 reflections with *I* > 2σ(*I*)
Graphite	*R*_int_ = 0.061
Detector resolution: 8.3 pixels mm^-1^	θ_max_ = 25.3°, θ_min_ = 1.2°
ω scans	*h* = −10→10
Absorption correction: multi-scan (*SADABS*; Sheldrick, 1996)	*k* = −14→14
*T*_min_ = 0.83, *T*_max_ = 0.95	*l* = −20→20
178384 measured reflections	

### Refinement

**Table:**

Refinement on *F*^2^	Primary atom site location: structure-invariant direct methods
Least-squares matrix: full	Secondary atom site location: difference Fourier map
*R*[*F*^2^ > 2σ(*F*^2^)] = 0.055	Hydrogen site location: inferred from neighbouring sites
*wR*(*F*^2^) = 0.152	H-atom parameters constrained
*S* = 1.18	*w* = 1/[σ^2^(*F*_o_^2^) + (0.0708*P*)^2^ + 4.8035*P*] where *P* = (*F*_o_^2^ + 2*F*_c_^2^)/3
6687 reflections	(Δ/σ)_max_ = 0.001
536 parameters	Δρ_max_ = 1.45 e Å^−^^3^
0 restraints	Δρ_min_ = −0.86 e Å^−^^3^

### Special details

**Table:**

Experimental. X-ray crystallographic data for I were collected from a single-crystal sample, which was mounted on a loop fiber. Data were collected using a Bruker smart diffractometer equiped with an *APEX* II CCD Detector, a graphite monochromator. The crystal-to-detector distance was 5.0 cm, and the data collection was carried out in 512 *x* 512 pixel mode. The initial unit-cell parameters were determined by a least-squares fit of the angular setting of strong reflections, collected by a 10.0 degree scan in 33 frames over four different parts of the reciprocal space (132 frames total).
Geometry. All e.s.d.'s (except the e.s.d. in the dihedral angle between two l.s. planes) are estimated using the full covariance matrix. The cell e.s.d.'s are taken into account individually in the estimation of e.s.d.'s in distances, angles and torsion angles; correlations between e.s.d.'s in cell parameters are only used when they are defined by crystal symmetry. An approximate (isotropic) treatment of cell e.s.d.'s is used for estimating e.s.d.'s involving l.s. planes.
Refinement. Refinement of *F*^2^ against ALL reflections. The weighted *R*-factor *wR* and goodness of fit *S* are based on *F*^2^, conventional *R*-factors *R* are based on *F*, with *F* set to zero for negative *F*^2^. The threshold expression of *F*^2^ > σ(*F*^2^) is used only for calculating *R*-factors(gt) *etc*. and is not relevant to the choice of reflections for refinement. *R*-factors based on *F*^2^ are statistically about twice as large as those based on *F*, and *R*- factors based on ALL data will be even larger.

### Fractional atomic coordinates and isotropic or equivalent isotropic displacement parameters (Å^2^)

**Table:**

	*x*	*y*	*z*	*U*_iso_*/*U*_eq_	
Ru	0.38640 (4)	0.62025 (3)	0.24834 (2)	0.03144 (14)	
S	−0.09857 (19)	0.94046 (14)	0.70014 (10)	0.0591 (4)	
N1	0.4231 (4)	0.7694 (3)	0.2119 (2)	0.0367 (8)	
N2	0.3529 (5)	0.6162 (4)	0.1294 (2)	0.0388 (9)	
N3	0.6085 (4)	0.5205 (3)	0.2332 (2)	0.0357 (8)	
N4	0.3658 (5)	0.4625 (3)	0.2763 (2)	0.0341 (8)	
N5	0.4022 (5)	0.6486 (3)	0.3666 (2)	0.0369 (9)	
N6	0.1613 (5)	0.7088 (3)	0.2710 (2)	0.0360 (9)	
N7	0.0887 (6)	0.8460 (4)	0.5781 (3)	0.0602 (13)	
C1	0.4583 (6)	0.8450 (4)	0.2582 (3)	0.0431 (11)	
H1	0.4678	0.8303	0.3130	0.052*	
C2	0.4806 (6)	0.9433 (4)	0.2279 (4)	0.0519 (14)	
H2	0.5066	0.9947	0.2615	0.062*	
C3	0.4652 (6)	0.9660 (5)	0.1489 (4)	0.0565 (15)	
H3	0.4791	1.0338	0.1275	0.068*	
C4	0.4294 (6)	0.8901 (5)	0.1012 (4)	0.0510 (13)	
H4	0.4183	0.9048	0.0464	0.061*	
C5	0.4095 (5)	0.7919 (4)	0.1334 (3)	0.0410 (11)	
C6	0.3705 (5)	0.7049 (4)	0.0877 (3)	0.0409 (11)	
C7	0.3534 (6)	0.7108 (5)	0.0063 (3)	0.0536 (14)	
H7	0.3704	0.7722	−0.0229	0.064*	
C8	0.3122 (7)	0.6279 (6)	−0.0310 (3)	0.0581 (15)	
H8	0.2978	0.6322	−0.0861	0.070*	
C9	0.2917 (7)	0.5380 (5)	0.0119 (3)	0.0524 (13)	
H9	0.2623	0.4800	−0.0132	0.063*	
C10	0.3144 (6)	0.5336 (4)	0.0915 (3)	0.0430 (11)	
H10	0.3027	0.4706	0.1209	0.052*	
C11	0.7259 (6)	0.5555 (4)	0.2073 (3)	0.0433 (11)	
H11	0.7092	0.6338	0.1968	0.052*	
C12	0.8704 (6)	0.4822 (5)	0.1952 (3)	0.0463 (12)	
H12	0.9506	0.5099	0.1760	0.056*	
C13	0.8972 (6)	0.3692 (5)	0.2112 (3)	0.0459 (12)	
H13	0.9962	0.3177	0.2042	0.055*	
C14	0.7764 (6)	0.3316 (4)	0.2379 (3)	0.0410 (11)	
H14	0.7922	0.2537	0.2498	0.049*	
C15	0.6340 (6)	0.4075 (4)	0.2469 (3)	0.0371 (10)	
C16	0.4958 (6)	0.3749 (4)	0.2704 (3)	0.0359 (10)	
C17	0.4963 (7)	0.2647 (4)	0.2856 (3)	0.0447 (12)	
H17	0.5869	0.2040	0.2786	0.054*	
C18	0.3620 (7)	0.2441 (4)	0.3113 (3)	0.0464 (12)	
H18	0.3604	0.1693	0.3234	0.056*	
C19	0.2330 (6)	0.3325 (4)	0.3189 (3)	0.0442 (12)	
H19	0.1402	0.3198	0.3361	0.053*	
C20	0.2374 (6)	0.4408 (4)	0.3015 (3)	0.0386 (10)	
H20	0.1469	0.5020	0.3075	0.046*	
C21	0.5229 (6)	0.6167 (4)	0.4138 (3)	0.0440 (11)	
H21	0.6173	0.5708	0.3936	0.053*	
C22	0.5166 (7)	0.6483 (5)	0.4923 (3)	0.0534 (14)	
H22	0.6053	0.6249	0.5246	0.064*	
C23	0.3801 (7)	0.7136 (5)	0.5214 (3)	0.0505 (13)	
H23	0.3741	0.7355	0.5746	0.061*	
C24	0.2514 (6)	0.7481 (4)	0.4746 (3)	0.0436 (12)	
C25	0.1014 (7)	0.8152 (4)	0.5000 (3)	0.0453 (12)	
C26	−0.0220 (7)	0.8445 (5)	0.4516 (4)	0.0527 (14)	
H26	−0.1182	0.8893	0.4705	0.063*	
C27	−0.0085 (6)	0.8087 (4)	0.3729 (3)	0.0450 (12)	
C28	−0.1299 (6)	0.8342 (5)	0.3191 (4)	0.0505 (13)	
H28	−0.2295	0.8775	0.3348	0.061*	
C29	−0.1060 (6)	0.7975 (5)	0.2446 (3)	0.0495 (13)	
H29	−0.1884	0.8139	0.2082	0.059*	
C30	0.0415 (6)	0.7351 (4)	0.2220 (3)	0.0437 (11)	
H30	0.0572	0.7103	0.1696	0.052*	
C31	0.1371 (6)	0.7452 (4)	0.3460 (3)	0.0400 (11)	
C32	0.2664 (6)	0.7137 (4)	0.3967 (3)	0.0405 (11)	
C33	0.0016 (7)	0.8891 (4)	0.6287 (3)	0.0510 (13)	
P1	1.0000	0.5000	0.5000	0.0572 (6)	
F11	0.9341 (5)	0.5008 (5)	0.4141 (3)	0.0975 (15)	
F12	0.8596 (5)	0.4634 (3)	0.5351 (3)	0.0808 (13)	
F13	0.9038 (5)	0.6276 (3)	0.5092 (3)	0.0901 (15)	
P2	0.5000	1.0000	0.5000	0.0427 (4)	
F21	0.4009 (6)	0.9596 (4)	0.5648 (3)	0.0951 (15)	
F22	0.6131 (6)	0.8810 (4)	0.5037 (5)	0.145 (3)	
F23	0.4083 (11)	0.9609 (7)	0.4380 (4)	0.177 (4)	
P3	0.17933 (17)	0.24155 (12)	0.02025 (9)	0.0502 (4)	
F31	0.1520 (7)	0.1887 (4)	0.1024 (3)	0.113 (2)	
F32	0.0179 (5)	0.2320 (4)	−0.0039 (5)	0.129 (2)	
F33	0.2061 (9)	0.2892 (4)	−0.0615 (3)	0.129 (2)	
F34	0.3376 (6)	0.2461 (4)	0.0479 (4)	0.1127 (19)	
F35	0.1002 (5)	0.3654 (3)	0.0503 (3)	0.0762 (11)	
F36	0.2583 (5)	0.1159 (3)	−0.0085 (2)	0.0712 (10)	
C40	0.8638 (9)	0.0429 (5)	0.1327 (4)	0.0609 (16)	
C41	1.0145 (9)	−0.0209 (6)	0.1065 (5)	0.0735 (19)	
H41A	1.0621	0.0296	0.0780	0.110*	
H41B	1.0056	−0.0805	0.0718	0.110*	
H41C	1.0779	−0.0550	0.1519	0.110*	
N8	0.7473 (8)	0.0933 (5)	0.1538 (4)	0.0753 (16)	

### Atomic displacement parameters (Å^2^)

**Table:**

	*U*^11^	*U*^22^	*U*^33^	*U*^12^	*U*^13^	*U*^23^
Ru	0.0366 (2)	0.0291 (2)	0.0311 (2)	−0.01360 (15)	0.00462 (14)	−0.00054 (14)
S	0.0651 (9)	0.0567 (9)	0.0595 (9)	−0.0231 (7)	0.0200 (7)	−0.0170 (7)
N1	0.038 (2)	0.0318 (19)	0.042 (2)	−0.0128 (16)	0.0057 (17)	−0.0009 (16)
N2	0.039 (2)	0.045 (2)	0.032 (2)	−0.0127 (18)	0.0064 (16)	−0.0004 (17)
N3	0.038 (2)	0.036 (2)	0.036 (2)	−0.0148 (17)	0.0040 (16)	−0.0035 (16)
N4	0.044 (2)	0.0305 (19)	0.0313 (19)	−0.0155 (17)	0.0008 (16)	−0.0018 (15)
N5	0.046 (2)	0.0314 (19)	0.039 (2)	−0.0206 (17)	0.0010 (18)	0.0002 (16)
N6	0.041 (2)	0.0303 (19)	0.039 (2)	−0.0144 (16)	0.0103 (17)	0.0019 (16)
N7	0.076 (3)	0.056 (3)	0.053 (3)	−0.025 (3)	0.022 (3)	−0.016 (2)
C1	0.044 (3)	0.033 (2)	0.053 (3)	−0.012 (2)	0.009 (2)	−0.002 (2)
C2	0.044 (3)	0.032 (3)	0.081 (4)	−0.013 (2)	0.013 (3)	−0.004 (3)
C3	0.048 (3)	0.039 (3)	0.083 (4)	−0.015 (2)	0.011 (3)	0.018 (3)
C4	0.047 (3)	0.048 (3)	0.061 (3)	−0.020 (2)	0.007 (3)	0.016 (3)
C5	0.035 (2)	0.040 (3)	0.047 (3)	−0.011 (2)	0.008 (2)	0.007 (2)
C6	0.034 (2)	0.049 (3)	0.041 (3)	−0.015 (2)	0.002 (2)	0.005 (2)
C7	0.050 (3)	0.073 (4)	0.041 (3)	−0.026 (3)	0.002 (2)	0.017 (3)
C8	0.055 (3)	0.092 (5)	0.035 (3)	−0.034 (3)	−0.004 (2)	0.003 (3)
C9	0.052 (3)	0.071 (4)	0.041 (3)	−0.028 (3)	0.002 (2)	−0.009 (3)
C10	0.046 (3)	0.046 (3)	0.041 (3)	−0.020 (2)	0.002 (2)	−0.005 (2)
C11	0.045 (3)	0.039 (3)	0.049 (3)	−0.016 (2)	0.004 (2)	−0.006 (2)
C12	0.038 (3)	0.055 (3)	0.050 (3)	−0.018 (2)	0.004 (2)	−0.010 (2)
C13	0.042 (3)	0.048 (3)	0.044 (3)	−0.007 (2)	0.000 (2)	−0.013 (2)
C14	0.046 (3)	0.037 (3)	0.039 (3)	−0.010 (2)	−0.001 (2)	−0.005 (2)
C15	0.045 (3)	0.038 (2)	0.030 (2)	−0.014 (2)	0.0004 (19)	−0.0069 (18)
C16	0.046 (3)	0.034 (2)	0.031 (2)	−0.017 (2)	0.0028 (19)	−0.0040 (18)
C17	0.057 (3)	0.032 (2)	0.046 (3)	−0.013 (2)	0.002 (2)	−0.004 (2)
C18	0.064 (3)	0.034 (3)	0.049 (3)	−0.026 (2)	0.003 (2)	0.001 (2)
C19	0.053 (3)	0.045 (3)	0.043 (3)	−0.027 (2)	0.004 (2)	0.002 (2)
C20	0.042 (3)	0.041 (3)	0.037 (2)	−0.018 (2)	0.003 (2)	−0.002 (2)
C21	0.049 (3)	0.041 (3)	0.047 (3)	−0.019 (2)	−0.002 (2)	−0.003 (2)
C22	0.065 (4)	0.051 (3)	0.047 (3)	−0.021 (3)	−0.009 (3)	0.002 (2)
C23	0.065 (4)	0.047 (3)	0.044 (3)	−0.023 (3)	0.005 (3)	−0.003 (2)
C24	0.057 (3)	0.037 (3)	0.043 (3)	−0.024 (2)	0.010 (2)	−0.003 (2)
C25	0.058 (3)	0.041 (3)	0.042 (3)	−0.023 (2)	0.014 (2)	−0.004 (2)
C26	0.056 (3)	0.042 (3)	0.061 (3)	−0.017 (3)	0.019 (3)	−0.003 (2)
C27	0.054 (3)	0.035 (3)	0.050 (3)	−0.020 (2)	0.012 (2)	−0.002 (2)
C28	0.041 (3)	0.047 (3)	0.064 (4)	−0.014 (2)	0.005 (2)	0.003 (3)
C29	0.043 (3)	0.047 (3)	0.059 (3)	−0.014 (2)	0.003 (2)	0.005 (2)
C30	0.044 (3)	0.042 (3)	0.048 (3)	−0.018 (2)	0.001 (2)	0.004 (2)
C31	0.047 (3)	0.030 (2)	0.048 (3)	−0.018 (2)	0.009 (2)	0.003 (2)
C32	0.056 (3)	0.029 (2)	0.042 (3)	−0.021 (2)	0.014 (2)	−0.0019 (19)
C33	0.063 (3)	0.039 (3)	0.056 (3)	−0.022 (3)	0.014 (3)	−0.004 (2)
P1	0.0660 (14)	0.0391 (10)	0.0700 (14)	−0.0214 (10)	0.0305 (11)	−0.0011 (9)
F11	0.081 (3)	0.127 (4)	0.083 (3)	−0.028 (3)	0.022 (2)	−0.014 (3)
F12	0.088 (3)	0.057 (2)	0.107 (3)	−0.037 (2)	0.051 (2)	−0.008 (2)
F13	0.103 (3)	0.045 (2)	0.123 (4)	−0.022 (2)	0.060 (3)	−0.005 (2)
P2	0.0473 (10)	0.0392 (9)	0.0432 (10)	−0.0152 (8)	0.0076 (8)	−0.0015 (8)
F21	0.112 (4)	0.068 (3)	0.104 (3)	−0.028 (2)	0.058 (3)	0.008 (2)
F22	0.096 (4)	0.054 (3)	0.268 (8)	−0.002 (2)	0.091 (5)	0.028 (4)
F23	0.297 (10)	0.212 (8)	0.097 (4)	−0.193 (8)	−0.071 (5)	0.027 (4)
P3	0.0517 (8)	0.0431 (7)	0.0573 (9)	−0.0161 (6)	0.0128 (7)	−0.0050 (6)
F31	0.170 (5)	0.068 (3)	0.100 (3)	−0.032 (3)	0.080 (4)	−0.011 (2)
F32	0.066 (3)	0.074 (3)	0.248 (8)	−0.018 (2)	−0.009 (4)	−0.045 (4)
F33	0.223 (7)	0.065 (3)	0.073 (3)	−0.002 (3)	0.052 (4)	0.006 (2)
F34	0.073 (3)	0.094 (3)	0.176 (6)	−0.031 (3)	−0.002 (3)	−0.034 (4)
F35	0.079 (3)	0.050 (2)	0.102 (3)	−0.0215 (18)	0.034 (2)	−0.0191 (19)
F36	0.078 (2)	0.0481 (19)	0.083 (3)	−0.0129 (17)	0.027 (2)	−0.0085 (18)
C40	0.076 (5)	0.045 (3)	0.071 (4)	−0.032 (3)	0.005 (3)	−0.004 (3)
C41	0.086 (5)	0.063 (4)	0.079 (5)	−0.033 (4)	0.021 (4)	−0.006 (3)
N8	0.077 (4)	0.059 (3)	0.094 (5)	−0.027 (3)	0.002 (3)	−0.015 (3)

### Geometric parameters (Å, °)

**Table:**

Ru—N1	2.056 (4)	C17—H17	0.95
Ru—N3	2.059 (4)	C18—C19	1.360 (8)
Ru—N2	2.059 (4)	C18—H18	0.95
Ru—N4	2.064 (4)	C19—C20	1.381 (7)
Ru—N6	2.065 (4)	C19—H19	0.95
Ru—N5	2.072 (4)	C20—H20	0.95
S—C33	1.554 (6)	C21—C22	1.403 (8)
N1—C1	1.350 (6)	C21—H21	0.95
N1—C5	1.363 (6)	C22—C23	1.369 (8)
N2—C6	1.345 (6)	C22—H22	0.95
N2—C10	1.357 (6)	C23—C24	1.379 (8)
N3—C11	1.339 (6)	C23—H23	0.95
N3—C15	1.365 (6)	C24—C32	1.401 (7)
N4—C20	1.344 (6)	C24—C25	1.448 (8)
N4—C16	1.358 (6)	C25—C26	1.356 (8)
N5—C21	1.326 (7)	C26—C27	1.419 (8)
N5—C32	1.370 (6)	C26—H26	0.95
N6—C30	1.337 (7)	C27—C28	1.402 (8)
N6—C31	1.365 (6)	C27—C31	1.412 (7)
N7—C33	1.197 (7)	C28—C29	1.356 (8)
N7—C25	1.391 (7)	C28—H28	0.95
C1—C2	1.382 (7)	C29—C30	1.398 (8)
C1—H1	0.95	C29—H29	0.95
C2—C3	1.371 (9)	C30—H30	0.95
C2—H2	0.95	C31—C32	1.420 (8)
C3—C4	1.370 (9)	P1—F13^i^	1.578 (4)
C3—H3	0.95	P1—F13	1.578 (4)
C4—C5	1.383 (7)	P1—F11	1.588 (5)
C4—H4	0.95	P1—F11^i^	1.588 (5)
C5—C6	1.472 (7)	P1—F12	1.591 (3)
C6—C7	1.396 (7)	P1—F12^i^	1.591 (3)
C7—C8	1.366 (9)	P2—F23^ii^	1.526 (5)
C7—H7	0.95	P2—F23	1.526 (5)
C8—C9	1.376 (9)	P2—F22^ii^	1.531 (4)
C8—H8	0.95	P2—F22	1.531 (4)
C9—C10	1.374 (7)	P2—F21	1.589 (4)
C9—H9	0.95	P2—F21^ii^	1.589 (4)
C10—H10	0.95	P3—F34	1.540 (5)
C11—C12	1.381 (7)	P3—F33	1.549 (5)
C11—H11	0.95	P3—F32	1.571 (5)
C12—C13	1.370 (8)	P3—F31	1.585 (5)
C12—H12	0.95	P3—F35	1.589 (4)
C13—C14	1.388 (8)	P3—F36	1.600 (4)
C13—H13	0.95	C40—N8	1.125 (9)
C14—C15	1.372 (7)	C40—C41	1.445 (10)
C14—H14	0.95	C41—H41a	0.98
C15—C16	1.484 (7)	C41—H41b	0.98
C16—C17	1.383 (7)	C41—H41c	0.98
C17—C18	1.390 (8)		
			
N1—Ru—N3	96.28 (15)	N4—C20—H20	118.9
N1—Ru—N2	79.03 (16)	C19—C20—H20	118.9
N3—Ru—N2	88.79 (16)	N5—C21—C22	122.5 (5)
N1—Ru—N4	174.09 (15)	N5—C21—H21	118.7
N3—Ru—N4	78.91 (15)	C22—C21—H21	118.7
N2—Ru—N4	97.32 (15)	C23—C22—C21	118.6 (5)
N1—Ru—N6	88.69 (15)	C23—C22—H22	120.7
N3—Ru—N6	174.60 (15)	C21—C22—H22	120.7
N2—Ru—N6	94.23 (16)	C22—C23—C24	120.8 (5)
N4—Ru—N6	96.25 (15)	C22—C23—H23	119.6
N1—Ru—N5	94.83 (15)	C24—C23—H23	119.6
N3—Ru—N5	97.41 (16)	C23—C24—C32	117.4 (5)
N2—Ru—N5	171.75 (16)	C23—C24—C25	125.1 (5)
N4—Ru—N5	89.23 (14)	C32—C24—C25	117.5 (5)
N6—Ru—N5	80.03 (16)	C26—C25—N7	121.2 (5)
C1—N1—C5	118.4 (4)	C26—C25—C24	122.4 (5)
C1—N1—RU	126.2 (3)	N7—C25—C24	116.4 (5)
C5—N1—RU	115.4 (3)	C25—C26—C27	120.5 (5)
C6—N2—C10	118.9 (4)	C25—C26—H26	119.7
C6—N2—RU	115.5 (3)	C27—C26—H26	119.7
C10—N2—RU	125.6 (3)	C28—C27—C31	117.4 (5)
C11—N3—C15	117.9 (4)	C28—C27—C26	124.4 (5)
C11—N3—RU	126.1 (3)	C31—C27—C26	118.3 (5)
C15—N3—RU	115.9 (3)	C29—C28—C27	120.3 (5)
C20—N4—C16	118.5 (4)	C29—C28—H28	119.9
C20—N4—RU	125.6 (3)	C27—C28—H28	119.9
C16—N4—RU	115.9 (3)	C28—C29—C30	119.2 (5)
C21—N5—C32	118.1 (4)	C28—C29—H29	120.4
C21—N5—RU	129.4 (4)	C30—C29—H29	120.4
C32—N5—RU	112.4 (3)	N6—C30—C29	122.9 (5)
C30—N6—C31	118.0 (4)	N6—C30—H30	118.6
C30—N6—RU	128.5 (3)	C29—C30—H30	118.6
C31—N6—RU	113.5 (3)	N6—C31—C27	122.3 (5)
C33—N7—C25	144.9 (6)	N6—C31—C32	116.4 (4)
N1—C1—C2	121.7 (5)	C27—C31—C32	121.3 (5)
N1—C1—H1	119.1	N5—C32—C24	122.5 (5)
C2—C1—H1	119.1	N5—C32—C31	117.5 (4)
C3—C2—C1	119.6 (6)	C24—C32—C31	119.9 (5)
C3—C2—H2	120.2	N7—C33—S	174.4 (6)
C1—C2—H2	120.2	F13^i^—P1—F13	180.0 (4)
C4—C3—C2	119.3 (5)	F13^i^—P1—F11	89.6 (3)
C4—C3—H3	120.4	F13—P1—F11	90.4 (3)
C2—C3—H3	120.4	F13^i^—P1—F11^i^	90.4 (3)
C3—C4—C5	119.7 (5)	F13—P1—F11^i^	89.6 (3)
C3—C4—H4	120.2	F11—P1—F11^i^	180.000 (2)
C5—C4—H4	120.2	F13^i^—P1—F12	90.9 (2)
N1—C5—C4	121.3 (5)	F13—P1—F12	89.1 (2)
N1—C5—C6	114.6 (4)	F11—P1—F12	90.3 (3)
C4—C5—C6	124.1 (5)	F11^i^—P1—F12	89.7 (3)
N2—C6—C7	120.8 (5)	F13^i^—P1—F12^i^	89.1 (2)
N2—C6—C5	115.5 (4)	F13—P1—F12^i^	90.9 (2)
C7—C6—C5	123.7 (5)	F11—P1—F12^i^	89.7 (3)
C8—C7—C6	119.7 (5)	F11^i^—P1—F12^i^	90.3 (3)
C8—C7—H7	120.2	F12—P1—F12^i^	180.000 (1)
C6—C7—H7	120.2	F23^ii^—P2—F23	180.0 (6)
C7—C8—C9	119.6 (5)	F23^ii^—P2—F22^ii^	89.3 (5)
C7—C8—H8	120.2	F23—P2—F22^ii^	90.7 (5)
C9—C8—H8	120.2	F23^ii^—P2—F22	90.7 (5)
C10—C9—C8	119.0 (5)	F23—P2—F22	89.3 (5)
C10—C9—H9	120.5	F22^ii^—P2—F22	180.0 (6)
C8—C9—H9	120.5	F23^ii^—P2—F21	91.9 (4)
N2—C10—C9	122.1 (5)	F23—P2—F21	88.1 (4)
N2—C10—H10	118.9	F22^ii^—P2—F21	91.9 (3)
C9—C10—H10	118.9	F22—P2—F21	88.1 (3)
N3—C11—C12	122.6 (5)	F23^ii^—P2—F21^ii^	88.1 (4)
N3—C11—H11	118.7	F23—P2—F21^ii^	91.9 (4)
C12—C11—H11	118.7	F22^ii^—P2—F21^ii^	88.1 (3)
C13—C12—C11	119.5 (5)	F22—P2—F21^ii^	91.9 (3)
C13—C12—H12	120.2	F21—P2—F21^ii^	180.000 (2)
C11—C12—H12	120.2	F34—P3—F33	90.6 (4)
C12—C13—C14	118.6 (5)	F34—P3—F32	176.7 (4)
C12—C13—H13	120.7	F33—P3—F32	92.6 (4)
C14—C13—H13	120.7	F34—P3—F31	90.3 (4)
C15—C14—C13	119.6 (5)	F33—P3—F31	178.0 (3)
C15—C14—H14	120.2	F32—P3—F31	86.6 (4)
C13—C14—H14	120.2	F34—P3—F35	91.2 (3)
N3—C15—C14	121.7 (5)	F33—P3—F35	90.9 (2)
N3—C15—C16	114.5 (4)	F32—P3—F35	89.8 (2)
C14—C15—C16	123.7 (4)	F31—P3—F35	90.9 (2)
N4—C16—C17	121.5 (5)	F34—P3—F36	88.8 (3)
N4—C16—C15	114.7 (4)	F33—P3—F36	90.2 (2)
C17—C16—C15	123.8 (5)	F32—P3—F36	90.1 (2)
C16—C17—C18	119.0 (5)	F31—P3—F36	88.0 (2)
C16—C17—H17	120.5	F35—P3—F36	178.9 (2)
C18—C17—H17	120.5	N8—C40—C41	179.0 (8)
C19—C18—C17	119.2 (5)	C40—C41—H41A	109.5
C19—C18—H18	120.4	C40—C41—H41B	109.5
C17—C18—H18	120.4	H41A—C41—H41B	109.5
C18—C19—C20	119.7 (5)	C40—C41—H41C	109.5
C18—C19—H19	120.2	H41A—C41—H41C	109.5
C20—C19—H19	120.2	H41B—C41—H41C	109.5
N4—C20—C19	122.1 (5)		
			
N3—RU—N1—C1	−92.9 (4)	C6—N2—C10—C9	−0.5 (8)
N2—RU—N1—C1	179.6 (4)	RU—N2—C10—C9	178.9 (4)
N6—RU—N1—C1	85.0 (4)	C8—C9—C10—N2	1.6 (9)
N5—RU—N1—C1	5.1 (4)	C15—N3—C11—C12	−0.8 (7)
N3—RU—N1—C5	87.3 (3)	RU—N3—C11—C12	−176.9 (4)
N2—RU—N1—C5	−0.2 (3)	N3—C11—C12—C13	−1.0 (8)
N6—RU—N1—C5	−94.8 (3)	C11—C12—C13—C14	1.2 (8)
N5—RU—N1—C5	−174.7 (3)	C12—C13—C14—C15	0.5 (7)
N1—RU—N2—C6	0.4 (3)	C11—N3—C15—C14	2.6 (7)
N3—RU—N2—C6	−96.2 (4)	RU—N3—C15—C14	179.0 (3)
N4—RU—N2—C6	−174.9 (3)	C11—N3—C15—C16	−176.1 (4)
N6—RU—N2—C6	88.3 (4)	RU—N3—C15—C16	0.3 (5)
N1—RU—N2—C10	−179.0 (4)	C13—C14—C15—N3	−2.5 (7)
N3—RU—N2—C10	84.4 (4)	C13—C14—C15—C16	176.1 (4)
N4—RU—N2—C10	5.7 (4)	C20—N4—C16—C17	3.4 (7)
N6—RU—N2—C10	−91.2 (4)	RU—N4—C16—C17	−178.3 (4)
N1—RU—N3—C11	0.1 (4)	C20—N4—C16—C15	−176.4 (4)
N2—RU—N3—C11	78.9 (4)	RU—N4—C16—C15	1.9 (5)
N4—RU—N3—C11	176.6 (4)	N3—C15—C16—N4	−1.4 (6)
N5—RU—N3—C11	−95.6 (4)	C14—C15—C16—N4	179.9 (4)
N1—RU—N3—C15	−176.0 (3)	N3—C15—C16—C17	178.8 (4)
N2—RU—N3—C15	−97.2 (3)	C14—C15—C16—C17	0.1 (7)
N4—RU—N3—C15	0.5 (3)	N4—C16—C17—C18	−3.2 (7)
N5—RU—N3—C15	88.3 (3)	C15—C16—C17—C18	176.5 (5)
N3—RU—N4—C20	176.8 (4)	C16—C17—C18—C19	1.6 (8)
N2—RU—N4—C20	−95.9 (4)	C17—C18—C19—C20	−0.3 (8)
N6—RU—N4—C20	−0.8 (4)	C16—N4—C20—C19	−2.1 (7)
N5—RU—N4—C20	79.1 (4)	RU—N4—C20—C19	179.9 (4)
N3—RU—N4—C16	−1.3 (3)	C18—C19—C20—N4	0.6 (8)
N2—RU—N4—C16	86.0 (3)	C32—N5—C21—C22	−0.8 (7)
N6—RU—N4—C16	−178.9 (3)	RU—N5—C21—C22	176.3 (4)
N5—RU—N4—C16	−99.0 (3)	N5—C21—C22—C23	0.6 (8)
N1—RU—N5—C21	−93.1 (4)	C21—C22—C23—C24	−0.2 (8)
N3—RU—N5—C21	3.9 (4)	C22—C23—C24—C32	−0.1 (8)
N4—RU—N5—C21	82.6 (4)	C22—C23—C24—C25	178.8 (5)
N6—RU—N5—C21	179.1 (4)	C33—N7—C25—C26	5.7 (12)
N1—RU—N5—C32	84.1 (3)	C33—N7—C25—C24	−172.5 (8)
N3—RU—N5—C32	−178.9 (3)	C23—C24—C25—C26	−178.5 (5)
N4—RU—N5—C32	−100.2 (3)	C32—C24—C25—C26	0.3 (7)
N6—RU—N5—C32	−3.7 (3)	C23—C24—C25—N7	−0.4 (8)
N1—RU—N6—C30	87.0 (4)	C32—C24—C25—N7	178.5 (4)
N2—RU—N6—C30	8.1 (4)	N7—C25—C26—C27	−177.5 (5)
N4—RU—N6—C30	−89.8 (4)	C24—C25—C26—C27	0.6 (8)
N5—RU—N6—C30	−177.9 (4)	C25—C26—C27—C28	179.5 (5)
N1—RU—N6—C31	−91.9 (3)	C25—C26—C27—C31	−2.0 (8)
N2—RU—N6—C31	−170.8 (3)	C31—C27—C28—C29	0.7 (8)
N4—RU—N6—C31	91.4 (3)	C26—C27—C28—C29	179.2 (5)
N5—RU—N6—C31	3.2 (3)	C27—C28—C29—C30	−0.8 (8)
C5—N1—C1—C2	−0.1 (7)	C31—N6—C30—C29	−0.2 (7)
RU—N1—C1—C2	−179.9 (4)	RU—N6—C30—C29	−179.0 (4)
N1—C1—C2—C3	0.8 (8)	C28—C29—C30—N6	0.5 (8)
C1—C2—C3—C4	−0.7 (8)	C30—N6—C31—C27	0.1 (6)
C2—C3—C4—C5	0.0 (8)	RU—N6—C31—C27	179.1 (3)
C1—N1—C5—C4	−0.7 (7)	C30—N6—C31—C32	178.8 (4)
RU—N1—C5—C4	179.2 (4)	RU—N6—C31—C32	−2.2 (5)
C1—N1—C5—C6	−179.8 (4)	C28—C27—C31—N6	−0.3 (7)
RU—N1—C5—C6	0.0 (5)	C26—C27—C31—N6	−178.9 (4)
C3—C4—C5—N1	0.7 (8)	C28—C27—C31—C32	−178.9 (4)
C3—C4—C5—C6	179.8 (5)	C26—C27—C31—C32	2.4 (7)
C10—N2—C6—C7	−1.6 (7)	C21—N5—C32—C24	0.5 (7)
RU—N2—C6—C7	178.9 (4)	RU—N5—C32—C24	−177.1 (3)
C10—N2—C6—C5	178.9 (4)	C21—N5—C32—C31	−178.7 (4)
RU—N2—C6—C5	−0.6 (5)	RU—N5—C32—C31	3.8 (5)
N1—C5—C6—N2	0.4 (6)	C23—C24—C32—N5	−0.1 (7)
C4—C5—C6—N2	−178.7 (5)	C25—C24—C32—N5	−179.0 (4)
N1—C5—C6—C7	−179.1 (5)	C23—C24—C32—C31	179.1 (4)
C4—C5—C6—C7	1.8 (8)	C25—C24—C32—C31	0.1 (7)
N2—C6—C7—C8	2.7 (8)	N6—C31—C32—N5	−1.1 (6)
C5—C6—C7—C8	−177.9 (5)	C27—C31—C32—N5	177.6 (4)
C6—C7—C8—C9	−1.6 (9)	N6—C31—C32—C24	179.8 (4)
C7—C8—C9—C10	−0.5 (9)	C27—C31—C32—C24	−1.5 (7)

Symmetry codes: (i) −*x*+2, −*y*+1, −*z*+1; (ii) −*x*+1, −*y*+2, −*z*+1.
